# Impact of residual skin lesions and previous biologic treatment failure on patient‐reported outcomes in patients with psoriasis receiving biologic treatment

**DOI:** 10.1111/1346-8138.17249

**Published:** 2024-04-25

**Authors:** Won Ji Song, Hyun‐Sun Yoon

**Affiliations:** ^1^ Department of Dermatology Seoul National University Hospital Seoul Korea; ^2^ Department of Dermatology Seoul National University College of Medicine Seoul Korea; ^3^ Department of Dermatology SMG‐SNU Boramae Medical Center Seoul Korea

**Keywords:** biologics, patient‐reported outcomes, psoriasis, quality of life, treatment failure

## Abstract

Recent advances in biologic treatments have made clear skin a realistic treatment goal for psoriasis. However, clear skin may not uniformly translate to an absence of impact on patients' quality of life. This retrospective observational study aimed to elucidate the factors influencing patient‐reported outcomes in patients with psoriasis who have demonstrated successful clinician‐reported outcomes on using biologics. A total of 96 patients who have achieved a ≥75% improvement in Psoriasis Area and Severity Index (PASI) scores with ≥6 months of biologic treatment were included. Their median PASI score was 0.4, with 37.5% having achieved PASI 100 (clear skin). Furthermore, 47.9% reported no impact of psoriasis on their quality of life (Dermatology Life Quality Index [DLQI] score 0 or 1), while 52.1% reported a negative impact (DLQI score ≥2). Notably, 28.1% of the participants had a history of biologic treatment failure, defined as the inability to achieve or sustain a 75% PASI improvement with the previously used biologic agent. Multivariable logistic regression analysis revealed a positive association between achieving PASI 100 and reporting no impact of psoriasis on quality of life (adjusted odds ratio [aOR] 3.88, 95% confidence interval [CI] 1.49–10.91, *P* = 0.007). Conversely, prior biologic treatment failure was negatively associated with reporting no impact of psoriasis on quality of life (aOR 0.13, 95% CI 0.02–0.65, *P* = 0.023). Furthermore, among patients with clear skin, those with experience of previous biologic treatment failure reported significantly lower quality of life than those without such experience (*P* = 0.033). In conclusion, minimal residual skin lesions and prior biologic treatment failure were associated with poorer patient‐reported outcomes in patients with psoriasis. Opting for a biologic agent with the highest predicted efficacy, rather than pursuing a “step‐up” approach with a higher possibility of treatment failure, may be a more suitable strategy in the biologic treatment of psoriasis.

## 1 INTRODUCTION

Psoriasis is a chronic inflammatory skin disease that affects approximately 2%–3% of the global population.^1^, ^2^ Characterized by erythematous, scaly plaques, and often accompanied by itching or discomfort, psoriasis can markedly impact both the physical and emotional well‐being of those affected.^3^, ^4^, ^5^, ^6^ Recent advances in biologic treatments have revolutionized psoriasis management, offering patients the potential for near or complete skin clearance.^7^, ^8^ These clinical improvements are paralleled by enhancements in patient‐reported outcome measures, including the Dermatology Life Quality Index (DLQI).^8^, ^9^, ^10^, ^11^, ^12^, ^13^, ^14^, ^15^, ^16^, ^17^

However, it is imperative to note that achieving clear skin does not guarantee an optimal quality of life for all patients. Several studies have revealed that approximately 20% of patients with clear skin still report a negative impact of psoriasis on their quality of life, as indicated by DLQI scores exceeding 1.^18^, ^19^ Moreover, among those with nearly clear skin, almost half reported a negative impact of the disease on their quality of life.^18^ These findings are consistent with those of several studies that identified a mismatch between clinician‐ and patient‐reported outcomes, suggesting that patients' perceptions of the impact of their condition may differ from clinicians' observations.^20^, ^21^, ^22^ Consequently, understanding factors associated with patient‐reported outcomes among patients with psoriasis is paramount for bridging the gap between the perspectives of clinicians and patients, especially in the current era of biologics where both clinician‐ and patient‐reported outcomes are considered crucial for achieving true treatment success.^22^, ^23^

In light of these considerations, the objective of our study was to investigate patient‐reported outcomes assessed using the DLQI in patients with chronic plaque psoriasis who achieved promising clinician‐reported outcomes following biologic treatments. Additionally, we explored the factors associated with patient‐reported outcomes to shed light on why achieving clear skin does not always equate to holistic well‐being.

## 2 METHODS

### 2.1 Design and protocol

This retrospective observational study was conducted at the Seoul Metropolitan Government, Seoul National University (SMG‐SNU) Boramae Medical Center, an academic hospital in Korea. Medical data were extracted from December 1, 2022, to May 31, 2023. The requirement for informed consent was waived by the Institutional Review Board (IRB) of the SMG‐SNU Boramae Medical Center after reviewing the study protocol (IRB No. 30‐2023‐77).

### 2.2 Study population and data acquisition

Patients aged ≥18 years with a diagnosis of chronic plaque psoriasis confirmed by a dermatologist and who responded to >6 months of current and continuous treatment with biologics at SMG‐SNU Boramae Medical Center were included in the study. Treatment response was defined as the achievement of a ≥75% improvement in the Psoriasis Area and Severity Index (PASI) score from baseline. Patients with records of DLQI scores, PASI scores, and location of residual lesions assessed on the same day of visit were considered eligible for the study. The DLQI is a one of the most widely used and validated tools for assessing the patient‐reported outcomes of patients with dermatological diseases.^24^ The DLQI consists of 10 questions, each of which can be scored on a scale of 0 (strongly disagree) to 3 (strongly agree). The total DLQI score, calculated by summing the scores of the 10 questions, ranges from 0 to 30, with higher scores representing greater impairment of quality of life. A DLQI score of 0 or 1 indicates no impact of the disease on the quality of life, whereas a DLQI score of >2 indicates a negative impact.^25^ Patients with records of DLQI scores included in this study were evaluated using a validated Korean version of the DLQI questionnaire.^26^ The following data were further collected from the patients' medical records: age, sex, body mass index (BMI), baseline PASI score, previous experience of biologic failure, duration of treatment with current biologics, total duration of treatment with any biologics, and comorbidities (psoriatic arthritis, diabetes mellitus, hypertension, dyslipidemia, and cardiovascular, pulmonary, hepatic, renal, or autoimmune diseases).

### 2.3 Statistical analysis

Data are presented as mean ± standard deviation (SD) for normally distributed continuous variables and as median values and interquartile ranges (IQRs) for non‐normally distributed continuous variables. Categorical variables are expressed as frequencies and percentages.

We conducted univariable and multivariable logistic regression analyses to identify factors associated with DLQI scores. The dependent variable was categorized as DLQI score of 0–1 (indicating no impact on quality of life) or ≥2 (indicating a negative impact on quality of life). All collected variables, except for body mass index with partially missing data, were used as independent variables.

To assess variations in PASI or DLQI scores between groups with and without previous biologic failure, we utilized the Wilcoxon rank‐sum test due to the non‐parametric distribution of these scores. The level of statistical significance was set at *P* = 0.05. Graph generation and statistical analyses were performed using R Statistical Software version 4.3.2 (R Foundation for Statistical Computing, Vienna, Austria).

## 3 RESULTS

### 3.1 Study participants

Overall, 96 patients who responded to biologic treatment were included in the study (Table 1). Their median PASI score was 0.4 (IQR 0.0–1.2), with 37.5% (36 patients) achieving PASI 100 (complete skin clearance). After excluding individuals achieving PASI 100, the median PASI score was 1.2 (IQR: 0.5–1.8).

**TABLE 1:** Clinical characteristics of patients included in the study.

Characteristics	Total *N* = 96	No impact on quality of life (DLQI 0–1) *N* = 46	Negative impact on quality of life (DLQI ≥2) *N* = 50
Age (year), mean ± SD	43.7 ± 12.5	42.6 ± 11.2	44.8 ± 13.5
Male patients, *n* (%)	73 (76.0)	37 (80.4)	36 (72.0)
Clear skin (PASI 100), *n* (%)	36 (37.5)	26 (54.2)	12 (24.0)
PASI score, median (IQR)	0.4 (0.0–1.2)	0.0 (0.0–0.6)	1.1 (0.2–1.9)
DLQI score, median (IQR)	2.0 (0.0–4.0)	0.0 (0.0–0.0)	4.0 (2.3–6.0)
Body mass index (kg/m^2^), mean ± SD	25.6 ± 3.9	26.1 ± 3.7	25.0 ± 4.1
Current biologic agent, *n* (%)			
Ustekinumab	20 (20.8)	8 (16.7)	12 (24.0)
Secukinumab	8 (8.3)	6 (12.5)	2 (4.0)
Ixekizumab	17 (17.7)	5 (10.4)	12 (24.0)
Guselkumab	25 (26.0)	15 (31.3)	11 (22.0)
Risankizumab	26 (27.1)	14 (29.2)	13 (26.0)
Duration of treatment with current biologics (week), mean ± SD	133.4 ± 90.0	134.1 ± 84.3	132.7 ± 95.8
Total duration of treatment with any biologics (week), mean ± SD	189.6 ± 117.1	178.4 ± 110.3	199.9 ± 123.3
Baseline PASI score, median (IQR)	12.0 (10.8–16.2)	12.0 (11.0–14.4)	12.2 (10.6–16.3)
Previous biologic failure, *n* (%)	27 (28.1)	7 (15.2)	20 (40.0)
Location of residual lesions^a^, *n* (%)			
Head and neck	39 (40.6)	14 (30.4)	25 (50.0)
Trunk	10 (10.4)	1 (2.2)	9 (18.0)
Upper extremities	14 (14.6)	6 (13.0)	8 (16.0)
Lower extremities	27 (28.1)	8 (17.4)	19 (38.0)
Residual lesion in the exposed area, *n* (%)	62 (64.6)	24 (52.2)	38 (76.0)
Comorbidities except PsA, *n* (%)	28 (29.2)	15 (32.6)	13 (26.0)
PsA, *n* (%)	4 (4.2)	2 (4.4)	2 (4.0)

Abbreviations: DLQI, Dermatology Life Quality Index; IQR, interquartile range, 25th–75th percentiles; PASI, Psoriasis Area and Severity Index; PsA, psoriatic arthritis; SD, standard deviation.

^a^ Several patients had residual lesions in multiple locations.

The study participants comprised 73 (76.0%) men, with a mean age of 43.7 years (SD: 12.5). The biologics used by the study participants were ustekinumab, secukinumab, ixekizumab, guselkumab, or risankizumab. The most commonly used biologic was risankizumab (*n* = 26 patients), followed by guselkumab (*n* = 25 patients). No patient was on concomitant systemic treatment. Among the 96 patients included, 28.1% (27 patients) had previous experience of biologic treatment failure, while the remaining 71.9% (69 patients) did not have an experience of biologic treatment failure.

Of the total participants, 47.9% (46 patients) reported no impact of psoriasis on their quality of life (DLQI score of 0 or 1), while 52.1% (50 patients) reported a negative impact (DLQI score of ≥2). The clinical characteristics of the study participants, categorized into one of the two DLQI groups, are presented in Table 1.

### 3.2 Results of the univariable and multivariable logistic regression analyses

In the univariable analysis and multivariable analyses, achieving PASI 100 showed a significant association with reporting no impact of psoriasis on quality of life (adjusted odds ratio [aOR] 3.88, 95% confidence interval [CI] 1.49–10.91, *P* = 0.007; Table 2). Having experience of previous biologic treatment failure, however, was associated with a lower likelihood of reporting no impact of psoriasis on quality of life (aOR 0.13, 95% CI 0.02–0.65, *P* = 0.023). In other words, patients with residual skin lesions and a history of previous biologic treatment failure were more likely to report a negative impact of psoriasis on their quality of life.

**TABLE 2:** Results of logistic regression analysis of factors associated with reporting no impact of psoriasis on quality of life.

Variables	Univariable		Multivariable	
	OR (95% CI)	*P* value	OR (95% CI)	*P* value
Age, per 1 year increase	0.99 (0.95–1.02)	0.455	0.98 (0.93–1.03)	0.399
Male patients	1.60 (0.62–4.28)	0.335	1.98 (0.67–6.25)	0.225
Clear skin (PASI 100)	3.46 (1.47–8.46)	0.005^*^	3.88 (1.49–10.91)	0.007^*^
Body mass index, per 1 kg/m^2^ increase^a^	0.99 (0.97–1.01)	0.433		
Current use of IL‐23 inhibitor (vs IL‐17 inhibitor)	1.24 (0.50–3.15)	0.649	0.97 (0.28–3.33)	0.966
Duration of treatment with current biologics, per 1‐week increase	1.00 (1.00–1.01)	0.939	1.00 (0.99–1.01)	0.360
Total duration of treatment with any biologics, per 1‐week increase	1.00 (1.00–1.01)	0.368	1.01 (1.00–1.02)	0.188
Baseline PASI score, per 1 increase	0.99 (0.93–1.05)	0.784	1.00 (0.93–1.07)	0.893
Residual lesion in the exposed area^b^	0.34 (0.14–0.81)	0.016^*^		
Previous biologic failure	0.27 (0.10–0.69)	0.009^*^	0.13 (0.02–0.65)	0.023^*^
Comorbidities including PsA	1.24 (0.52–3.00)	0.623	1.33 (0.45–4.03)	0.604

Abbreviations: CI, confidence interval; IL, interleukin; OR, odds ratio; PASI, Psoriasis Area and Severity Index; PsA, psoriatic arthritis.

^*^ These values are statistically significant (*p* < 0.05).

^a^ Not included in the multivariable analysis due to missing data.

^b^ Not included in the multivariable analysis due to multicollinearity with the variable “clear skin”.

Due to the high multicollinearity between the variables “residual lesion in the exposed area” and “clear skin”, we conducted a separate multivariable analysis that included the variable “residual lesion in the exposed area” while excluding the variable “clear skin.” The presence of residual lesions in the exposed area was likewise associated with patients reporting a negative impact of psoriasis on their quality of life (aOR 0.33, 95% CI 0.12–0.85, *P* = 0.024; Table S1).

### 3.3 Quality of life based on experience of previous biologic treatment failure

**FIGURE 1:**
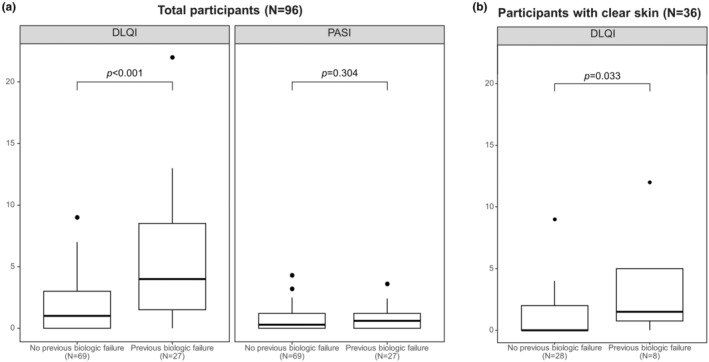
Comparison of treatment outcomes according to the previous biologic treatment failure. (a) Comparison of Dermatology Life Quality Index (DLQI) and Psoriasis Area and Severity Index (PASI) scores between patients with and without previous biologic treatment failure, using the Wilcoxon rank‐sum test. (b) Comparison of DLQI scores in patients who have achieved PASI 100 (clear skin) with biologic treatment based on their experience of previous biologic treatment failure, using the Wilcoxon rank‐sum test.

While the median PASI score did not show a significant difference based on the experience of previous biologic treatment failure (0.6, IQR 0.0–1.2 vs 0.3, IQR 0.0–1.2, *P* = 0.304), the median DLQI score was significantly higher in patients with such experiences compared to those without (4, IQR 1.5–8.5 vs 1, IQR 0–3, *P* < 0.001) (Figure 1a).

Among patients with clear skin, those with a history of biologic treatment failure also showed significantly higher median DLQI score compared to those without such experience (2, IQR 0.75–5 vs 0, IQR 0–2, *P* = 0.033) (Figure 1b).

## 4 DISCUSSION

Our study shows that even minimal residual skin lesions can significantly influence patients' perceptions of the impact of psoriasis. Despite achieving a median PASI score as low as 0.4 with biologics, over half of the study participants reported a negative impact of psoriasis, indicating a notable discordance between clinician‐assessed outcomes and patient experiences. In line with our findings, Takeshita et al. demonstrated that individuals with clear versus almost clear skin, under any systemic therapy or phototherapy for psoriasis, were more likely to report no impact on their quality of life. Our study extends this observation to patients undergoing biologic treatments. Furthermore, our findings highlight the impact of previous biologic treatment failure on patients' quality of life. Although the median PASI score did not differ based on previous treatment failure, the median DLQI score was significantly higher among those with such experiences. Even among patients with clear skin, DLQI scores varied significantly based on their history of biologic treatment failure. This suggests that, regardless of an excellent response to the current biologic agent, prior failure of biologic treatment leaves a substantial effect on patients' quality of life.

The majority (85.2%) of instances of biologic treatment failure were categorized as secondary failure, defined as a drop below 75% PASI improvement (PASI75) after an initial achievement of PASI75. This suggests that prior experiences of biologic failure, signaling a decline in drug efficacy, might have prompted patients to question the persistency of the excellent efficacy of the currently used biologic agent. According to a survey conducted by the National Psoriasis Foundation, 88% of young patients with psoriasis expressed concerns about the disease worsening.^5^ Our study findings indicate that such apprehensions may persist even in patients who have achieved clear skin through biologic treatment.

To the best of our knowledge, no prior study has specifically investigated the discordance in patient‐reported outcomes based on experiences of biologic treatment failure. However, randomized controlled trials of interleukin (IL)‐17/IL‐23 inhibitors have indicated a tendency toward better patient‐reported outcomes in trials with a higher percentage of biologic‐naïve patients without a history of biologic failure. For instance, a study on guselkumab, with 78% biologic‐naïve patients, reported a DLQI score 0/1 rate of 60.9% after 6 months of treatment, whereas another guselkumab study, mostly including those with a history of biologic failure, reported a lower DLQI score 0/1 rate of 38.8%.^27^, ^28^, ^29^, ^30^, ^31^

The participants in our study showed a robust clinical response to biologic treatment, evidenced by a median PASI score of 0.4. Even after excluding individuals who achieved an absolute PASI score of 0, the median PASI score remained low at 1.2. This indicates that even minimal residual skin lesions were associated with poorer patient‐reported outcomes. We found that the distribution of residual skin lesions across different body locations was diverse, with the head and neck being the most common. This finding aligns with previous research, which suggested that skin lesions in specific areas that are often under‐represented in the PASI score, such as the scalp or neck, can exert a significant impact on patient‐reported outcomes.^32^, ^33^ While our study did not find an independent association of residual lesions in the head and neck region alone with poorer patient‐reported outcomes, the presence of residual lesions in exposed areas combined was significantly associated with poorer patient‐reported outcomes, consistent with prior research findings.^34^

Our analysis did not establish a significant association between DLQI scores and age, sex, or comorbidities, in contrast to some previous studies.^35^, ^36^, ^37^ This difference may be attributed to variations in study populations and treatment modalities. Previous studies on patient‐reported outcomes in psoriasis often included topical therapies, conventional systemic therapies, or TNF‐α inhibitors, whereas our study exclusively focused on patients undergoing treatment with IL‐17/IL‐23 inhibitors, one of the most widely prescribed classes of biologics in current practice.

Our study has some limitations that should be acknowledged. First, the retrospective nature of data collection and small sample size warrant caution in generalizing the study findings. Second, since DLQI scores were assessed cross‐sectionally, we refrained from drawing firm conclusions about the temporal evolution of patient‐reported outcomes. Lastly, while we lacked information on individual psychological conditions and socioeconomic status, a review of medical records indicated no patients simultaneously receiving psychiatric care. Additionally, all patients in our study were covered by health insurance, mitigating the financial burden of psoriasis treatment. In fact, the out‐of‐pocket expenses for psoriasis treatment for our study participants were typically less than $800 annually, suggesting that in this particular context, the socioeconomic status of patients may not have substantially influenced the outcomes.

In conclusion, our study demonstrates that minimal residual skin lesions and the experience of previous biologic treatment failure are negatively associated with patient‐reported outcomes in patients with psoriasis treated with biologics. To achieve genuine treatment success aligned with patient perspectives, it is crucial not only to target PASI 100 but also to opt for a biologic agent with the highest predicted efficacy. This approach is preferable over a “step‐up” strategy, which carries a higher risk of treatment failure.

## CONFLICT OF INTEREST STATEMENT

Authors declare no conflict of interests for this article.

## ETHICS STATEMENT

Reviewed and approved by the Institutional Review Board (IRB) of SMG‐SNU Boramae Medical Center (IRB No. 30–2023‐77). The requirement for obtaining informed consent was waived by the IRB.

## Supporting information


Supporting Information Table S1.

## Data Availability

The data underlying this article will be shared on reasonable request to the corresponding author.
