# Supplementary material for: Impact of residual skin lesions and previous biologic treatment failure on patient‐reported outcomes in patients with psoriasis receiving biologic treatment
Source: J Dermatol. 2024 Apr 25;51(6):772–8. doi: 10.1111/1346-8138.17249 (PMC11483962; doi:10.1111/1346-8138.17249)
Supplement: Supplementary file 1 — Supporting Information Table S1. [file JDE-51--s001.docx]

**Supplementary Table 1. Results of multivariable logistic regression analysis including the variable “residual lesion in the exposed area” and excluding the variable “clear skin” due to multicollinearity.**

| **Variables** | **Multivariable** | |
| --- | --- | --- |
|  | **OR (95% CI)** | ***P*-value** |
| **Age**, per 1 year increase | 0.98 (0.94–1.02) | 0.400 |
| **Male patients** | 1.80 (0.62–5.49) | 0.286 |
| **Body mass index**, per 1kg/m^2^ increase^*^ |  |  |
| **Current use of IL-23 inhibitor** (vs. IL-17 inhibitor) | 0.88 (0.26–2.90) | 0.829 |
| **Duration of treatment with current biologics**, per 1-week increase | 1.00 (0.99–1.00) | 0.351 |
| **Total duration of treatment with any biologics**, per 1-week increase | 1.01 (1.00–1.01) | 0.207 |
| **Baseline PASI score**, per 1 increase | 1.00 (0.93–1.06) | 0.852 |
| **Residual lesion in the exposed area** | 0.33 (0.12–0.85) | 0.024^†^ |
| **Previous biologic failure** | 0.13 (0.02–0.63) | 0.020^†^ |
| **Comorbidities including PsA** | 1.37 (0.46–4.10) | 0.571 |

*OR*, Odds ratio; *CI*, Confidence interval; *IL*, Interleukin; *PASI*, Psoriasis Area and Severity Index; *PsA*, Psoriatic arthritis.

^*^Not included in the multivariable analysis due to missing data.

^†^These values are statistically significant (*p*<0.05).
